# Supraphysiological ascorbic acid infusion attenuates cerebrovascular dysfunction in middle‐aged and older adults

**DOI:** 10.14814/phy2.71120

**Published:** 2026-10-04

**Authors:** Theodore M. DeConne, Faria Sanjana, Elizabeth M. Habash, Kevin P. Decker, Wendy Nichols, Joshua C. Hobson, Nicholas A. Rizzi, Christopher R. Martens

**Affiliations:** ^1^ Department of Kinesiology and Applied Physiology University of Delaware Newark Delaware USA

**Keywords:** aging, antioxidant, cerebral blood flow, cerebrovascular function, endothelial function

## Abstract

Cerebrovascular endothelial function can be assessed by measuring the shear‐mediated dilation or cerebrovascular reactivity (CVR) of the internal carotid artery (ICA) in response to transient hypercapnia (30 s). ICA CVR is blunted with aging; however, it is not known whether age‐related reductions in ICA CVR are influenced by antioxidant‐responsive mechanisms. The purpose of this study was to determine whether infusion of the antioxidant, ascorbic acid (AA) restores ICA CVR in middle‐aged and older (MA/O) adults. We also examined the effect of AA on middle cerebral artery (MCA) CVR to sustained (3‐min) hypercapnia. We hypothesized that MA/O adults would exhibit lower cerebrovascular function compared to young (Y) adults, and that infusion of AA would improve cerebrovascular function in MA/O adults, whereas infusion of saline (SAL) would not. The primary analytic sample included nine Y adults (25 ± 2 years) and nineteen (65 ± 6 years) MA/O adults, with analyzable data varying by outcome. ICA CVR was measured using high‐resolution duplex ultrasound, and MCA CVR was measured using transcranial Doppler ultrasound. AA infusion improved ICA CVR and MCA CVR in MA/O adults but had no effect in Y adults. These findings suggest that age‐related impairments in cerebrovascular function may be partly attributable to mechanisms responsive to acute, supraphysiological AA administration.

## Graphical abstract

Young (Y) and middle‐aged and older (MA/O) community‐dwelling adults were recruited and randomized to acute ascorbic acid (AA) or saline (SAL) infusions, on separate days. Cerebrovascular reactivity (CVR) to transient hypercapnia (30 s) in the internal carotid artery (ICA), or sustained hypercapnia (3 min) in the middle cerebral artery (MCA), was measured before and after each infusion. AA infusion restored ICA and MCA CVR in MA/O, but not Y adults.

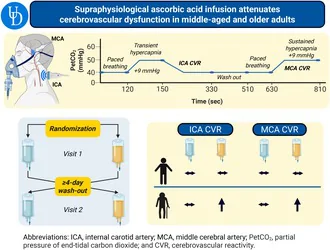

## 1 INTRODUCTION

Age‐related reductions in total cerebral blood flow (TCBF) and cerebrovascular reactivity (CVR) are independently associated with a higher risk for dementia, even after adjusting for other risk factors (Alwatban et al., 2019; Aslanyan et al., 2024; Duan et al., 2021; Peng et al., 2018; Sur et al., 2020; Wang et al., 2023; Weijs et al., 2023; Wolters et al., 2017). Concurrent with the decrease in cerebrovascular function, vascular aging is associated with an increase in oxidative stress (Martens & Seals, 2016; Mutlu‐Türkoğlu et al., 2003; Pinchuk et al., 2019; Seals et al., 2011). Oxidative stress occurs when there is an imbalance between reactive oxygen species and endogenous antioxidants (Sies, 1997; Verhaegen et al., 2022), thereby impairing vascular function, in part, by reducing nitric oxide (NO) bioavailability (Higashi et al., 2012; Martens & Seals, 2016; Seals et al., 2011; Tan et al., 2018; Ungvari, Kaley, et al., 2018; Ungvari, Tarantini, et al., 2018). Ascorbic acid (AA) is a potent antioxidant commonly used to “pharmaco‐dissect” the effect of oxidative stress on age‐related peripheral vascular dysfunction, including brachial (Moreau et al., 2013; Pierce et al., 2009; Rossman et al., 2018; Walker et al., 2012) and femoral (Jablonski et al., 2007; Moreau et al., 2005) artery endothelial function, and forearm microvascular (Jablonski et al., 2013) function in middle‐aged and older adults (MA/O).

Previous studies have demonstrated the effects of transient (30 s) hypercapnia on vasodilation of the ICA as a means of studying shear‐mediated endothelial function (Hoiland et al., 2017; Hoiland et al., 2022; Iwamoto et al., 2018), and a recent unblinded study implementing sustained (4‐min) hypercapnia by Freeberg et al., 2025 demonstrated that AA increased internal carotid artery CVR in both older men and women. However, the mechanisms through which aging impairs ICA shear‐mediated endothelial function in humans have not been fully elucidated. Although oxidative stress has been proposed as a potential contributor to age‐related vascular dysfunction, direct evidence for its role in cerebrovascular endothelial function is limited. Therefore, the purpose of this study was to determine whether acute supraphysiological AA infusion augments ICA shear‐mediated dilation in MA/O adults in a randomized, cross‐over, double‐blind, placebo‐controlled intervention study. We also examined the effects on total cerebral blood flow (TCBF), CVR to sustained (3‐min) hypercapnia in the middle cerebral artery (MCA), and peripheral endothelial function using flow‐mediated dilation (FMD). To do this, we infused AA in young (Y) and MA/O adults to “pharmaco‐dissect” potential age‐related mechanisms of cerebrovascular dysfunction. We hypothesized that cerebrovascular function would be lower in MA/O compared to Y adults, and that compared to the control infusion of saline (SAL), infusion of AA would improve cerebrovascular function only in MA/O adults.

## 2 MATERIALS AND METHODS

### 2.1 Participant screening

Generally healthy young (Y; aged 18–29 years) and middle‐aged and older adults (MA/O; aged 55–79 years) were recruited from Newark, Delaware and surrounding areas through flyers, phone, email, and letters. Participants reported no systemic medical illness (e.g., cardiovascular disease, cancer), major psychiatric disorders (e.g., schizophrenia), or neurological or autoimmune conditions (e.g., Parkinson's disease, multiple sclerosis, Type I diabetes, etc.). Participants were also excluded if they were currently smoking, pregnant or breast‐feeding, had abnormal clinical blood markers, took medication likely to affect central nervous system function, did not have a suitable temporal window for transcranial Doppler (TCD) ultrasound, or had a body mass index (BMI) ≥40 kg/m^2^.

### 2.2 Ethics statement

All procedures and protocols used were consistent with the standards set by the latest revisions of the Declaration of Helsinki (ClinicalTrials.gov ID: NCT03775382) and were approved by the University of Delaware Institutional Review Board (1138735). The study rationale, procedures, risks, and benefits were explained to the participants, and written informed consent was obtained prior to enrollment.

### 2.3 Clinical blood chemistries, resting blood pressure, and cognitive function

Clinical blood chemistries, including a comprehensive metabolic panel, complete lipid panel, and complete blood cell count, were measured by Quest Diagnostics and reviewed by a licensed Nurse Practitioner employed by the University of Delaware Nurse Managed Primary Care Center for determining eligibility. Seated blood pressure was obtained during screening and after each study visit in the non‐dominant arm following 10 min of quiet rest in a dimly lit room. Measurements were taken in 2 min intervals until three measurements were obtained within 5 mmHg of one another and then averaged. Global cognitive function and total recall were assessed during a separate study visit using the NIH Toolbox (Version 11, Total Composite Fully Corrected T‐Score) (Weintraub et al., 2013) and California Verbal Learning Test III (Total Recall Index Score) (Delis et al., 2022). Participants were encouraged to consume a snack prior to the cognitive exams.

### 2.4 Study design

Participants visited the laboratory twice for vascular testing, each time under a different condition: once with AA and once with SAL intravenous infusion (Figure S1). The order of these conditions was determined by a randomization schedule, with a minimum 4‐day washout between infusion visits. This washout period should be sufficient to ensure plasma AA returns to pre‐infusion concentration, as multiple studies have shown AA concentrations return to baseline within 24 h following intravenous infusion (Chen et al., 2022; Padayatty et al., 2004). Participants were asked to fast for ≥12 h, refrain from alcohol and vigorous aerobic exercise for 24 h, and refrain from over‐the‐counter medications or prescription cardiovascular‐acting medications for 48 h prior to each study visit.

### 2.5 Ascorbic acid and saline infusions

During the AA condition, USP‐grade ascorbic acid (500 mg/mL) obtained from individually packaged sterile dispensing vials (Saveway Compounding Pharmacy, Newark, DE) was infused into the antecubital vein using an intravenous infusion pump. The nurse administered a priming bolus of 0.06 g AA per kg fat‐free mass (determined by dual‐energy X‐ray absorptiometry [DXA] and/or bioelectrical impedance) dissolved in 100 mL of SAL for 20 min followed by a drip‐infusion of 0.02 g AA per kg fat‐free mass dissolved in 30 mL of SAL, as previously described (Eskurza et al., 2004). The drip infusion was maintained until the end of the study visit (Figure S1). Blood was obtained immediately before and after the infusion for analysis of AA concentration. The same procedures were performed during the SAL condition.

### 2.6 Ascorbic acid measurement

Serum AA was measured in an analytic sample before and after each infusion during each visit using ELISAs performed by the Biomarker Analytical Core at the Wake Forest University School of Medicine. Because the majority of samples from the present cohort were lost due to a freezer malfunction, serum AA was measured in three MA/O adults from this cohort and two Y adults (age = 21 ± 2 years; 50% female, BMI = 22.75 ± 2.19 kg/m^2^; systolic blood pressure = 109 ± 7; diastolic blood pressure = 67 ± 13; and heart rate = 65 ± 16) recruited for a small independent study who underwent the same AA infusion protocol.

### 2.7 Vascular function

Measures of cerebrovascular and peripheral vascular function were measured before and after the infusion of AA or SAL. Researchers, participants, and technicians were blinded to the intervention during the measurement and analysis of each vascular outcome.

### 2.8 Cerebrovascular reactivity to transient and sustained hypercapnia

ICA CVR was measured in response to transient hypercapnia, while MCA CVR was measured in response to sustained hypercapnia using a dynamically controlled prospective end‐tidal gas control system (RespirAct® RA‐MR™, Thornhill Medical, Toronto, ON, Canada). The transient hypercapnia protocol was performed before the sustained hypercapnia protocol, separated by a 3‐min washout. Beat‐by‐beat finger arterial pressure and heart rate (HR) were collected (Finapress® NOVA, Finapress Medical Systems). Brachial mean arterial pressure (MAP) was reconstructed from beat‐to‐beat finger arterial pressure. PowerLab (ADInstruments, PL3516 PowerLab 16/35) and LabChart (ADInstruments, #v8.1.13) were used to collect MCA velocity (MCA_V_) and beat‐to‐beat MAP and HR during the transient and sustained hypercapnic stimuli. Average beat‐to‐beat MCA_V_, MAP, and HR during each stimulus were extracted in LabChart using either the 3‐lead ECG or finger arterial pressure waveform to estimate each cardiac cycle. Participant end‐tidal partial pressure of CO_2_ (P_ET_CO_2_) was obtained from the RespirAct® throughout the entire protocol. Participants were guided by a metronome to breathe at a rate of 20 breaths per minute at baseline (2 min) and during the transient and sustained hypercapnia protocols. Following the paced‐breathing baseline period, P_ET_CO_2_ was rapidly increased by +9 mmHg above baseline for 30‐s (transient) or 3‐min (sustained) to investigate the respective shear‐mediated and CO_2_ vasodilatory responses (Hoiland et al., 2017).

Microsoft Excel (Version 2212) was used to plot and analyze data. The reconstructed beat‐to‐beat brachial arterial pressure was corrected for the baseline MAP by adjusting the baseline MAP for the beat‐to‐beat change in MAP during each of the protocols. The absolute change in reconstructed brachial MAP, HR, and P_ET_CO_2_ was calculated by taking the respective average value at baseline and subtracting it from the respective average value during the hyperemia period following the transient hypercapnia stimulus or the average value during the entire sustained hypercapnic stimulus.

### 2.9 Internal carotid artery cerebrovascular reactivity to transient hypercapnia

Cerebrovascular endothelial function was assessed by measuring the ICA vasodilatory response to transient hypercapnia. Following the paced‐breathing baseline period, P_ET_CO_2_ was rapidly increased by +9 mmHg above baseline for 30 s (transient) to investigate the shear‐mediated vasodilatory responses (Hoiland et al., 2017). The ICA was imaged using high‐resolution duplex ultrasound with a 10 MHz linear array transducer as previously described (Hoiland et al., 2017). All measures were made distal to the carotid bulb (to avoid measuring turbulent flow) at an insonation angle of 60°. ICA ultrasound files were analyzed using Cardiovascular Suite (Quipu, Pisa, Italy). ICA diameter, velocity, and shear rate data were then plotted using Microsoft Excel (Version 2212). ICA CVR to transient hypercapnia is hereafter referred to as “ICA CVR”. ICA diameter and blood flow velocity were measured at baseline, during the transient hypercapnia stimulus, and for 2 min following the transient hypercapnia stimulus (hyperemia period). CVR to transient hypercapnia was calculated as the percentage change in ICA diameter from the peak during the 2‐min recovery period following the transient hypercapnic stimulus to the average baseline ICA diameter. During each scan, anatomical landmarks were noted in the case report form. We aligned the ICA imaging location of subsequent measurements by reviewing the first ICA image and any landmarks noted in the case report form.

### 2.10 Middle cerebral artery cerebrovascular reactivity to sustained hypercapnia

MCA_V_ was measured using a 2‐MHz TCD ultrasound probe (Transcranial Ultrasound System, Spencer Technologies), positioned over the temporal window as previously described (Edwards et al., 2004; Zuj et al., 2012). MCA CVR to sustained hypercapnia was assessed by measuring the change in blood velocity of the intracranial blood vessel (MCA_V_). MCA_V_ was also normalized to beat‐to‐beat MAP for the calculation of MCA cerebrovascular conductance (MCA_CVC_) and MCA_CVC_ CVR. MCA CVR was calculated as the percentage change in MCA_V_ and MCA_CVC_ from the average baseline value to the peak during the 3‐min sustained hypercapnia stimulus and was then normalized to the absolute change in P_ET_CO_2_. MCA_V_ and MCA_CVC_ CVR are also reported as absolute change. MCA CVR to sustained hypercapnia is hereafter referred to as “MCA CVR.” The TCD probe was fixed in position using a headset and was not removed during the visit. Additionally, insonation depth, power, and head side were noted and kept consistent between infusions and visits. If an appropriate MCAv signal could not be located using these parameters, then we implemented the smallest adjustment possible. We also compared TCD depth and power at each visit and timepoint and included those data in the Supplemental Materials (Figure S2).

### 2.11 Total cerebral blood flow

TCBF was measured using high‐resolution duplex ultrasound with a 10 MHz linear array transducer (Logic E, GE Healthcare, Waukesha, Wisconsin). The internal carotid artery (ICA) and vertebral artery (VA) were imaged unilaterally for 1 min. The ultrasound files were analyzed using Cardiovascular Suite (Quipu, Pisa, Italy) and Microsoft Excel (Version 2212). The ICA and VA diameters (cm) and velocities (cm/s) derived from Cardiovascular Suite were averaged and then entered in Excel and converted to mm for the calculation of cross‐sectional area (cm^2^, see Equation 1 below). Flow rate for each vessel was then calculated from the product of the cross‐sectional area and average velocity (cm^3^/s) and converted to mL/min by multiplying by 60. TCBF was calculated as the sum of ICA and VA flow rate and then multiplied by two (mL/min). This approach assumes approximate bilateral symmetry in ICA and VA flow.

(1) $$\text{Cross sectional area}=\pi \times {\left[\mathrm{Avg}\;\text{Diameter}\;\left(\mathrm{cm}\right)/2\right]}^{2}$$

### 2.12 Flow‐mediated dilation

Peripheral vascular endothelial function was assessed using FMD in the participants' brachial artery (BA) of the dominant arm in a fully extended, 90‐degree position with blood vessel function examined using a LOGIQ e portable ultrasound (GE Healthcare, Chicago, IL). As previously described (Thijssen et al., 2011), the FMD test compared BA diameter from a 1‐min baseline period to the maximum BA diameter post‐occlusion of the forearm (directly below the elbow) to 220 mmHg using the Rapid Cuff Inflation System (Hokanson, Bellevue, WA). The hyperemia response was measured for 2 min after the occlusion period. The change in BA diameter, velocity, blood flow, and shear stress were calculated using FMD Studio in Cardiovascular Suite (Quipu, Pisa, Italy). Blood flow area under the curve (AUC), employed for the assessment of microvascular function, was calculated as the sum during the entire 2 min of post‐cuff occlusion, whereas shear rate AUC, employed to determine the stimulus for BA dilation, was calculated to the time point of peak dilation during the 2 min of post‐cuff occlusion. Peripheral vascular function was calculated as a percentage and absolute change in diameter from the average during baseline to peak during hyperemia. FMD as a percentage was normalized for the shear‐rate stimulus.

### 2.13 Statistics

To assess data quality for any inconsistencies or problematic data, descriptive statistics were first performed for each variable. Any non‐physiological or impossible values in the dataset (e.g., negative mean arterial pressure) were cross‐referenced with existing paper records or raw data files and corrected. Values that could not be corrected were omitted, unless there was no clear justification to exclude them, in which case they were retained in the analysis. Differences in participant characteristics were tested using unpaired Mann–Whitney test. The non‐parametric Mann–Whitney test was performed because of the small sample sizes in each age group (Y = 9 and MA/O = 19) (Smalheiser, 2017). Although the Mann–Whitney test compares rank differences between groups, participant characteristics are presented as mean ± SD for ease of interpretation. Due to technical difficulties with imaging or analyzing the peripheral or cerebral vessels, some participants had to be removed from each analysis. Our primary outcomes were the CVR measurements in the ICA in response to transient hypercapnia and MCA in response to sustained hypercapnia, thus the analytic counts for these measures are detailed in Tables S1 and S2, respectively.

As we initially hypothesized that older adults would exhibit lower vascular function, and that AA, but not SAL infusion, would augment vascular function in MA/O but not Y adults, Fisher's Least Significant Difference tests were used to evaluate pre‐specified, hypothesis‐driven contrasts. Specifically, these analyses were used to (1) assess whether pre‐infusion vascular function differed by age group and (2) test whether infusion condition affected vascular function within each age group. Two‐way ANOVA models with factors for age group and time point, conducted separately for the AA and SAL visits, were used to structure the data; interaction terms were examined (age group × time) but were not the primary basis for inference, as the study hypotheses were expressed in terms of specific a priori contrasts rather than omnibus interaction effects. Given that the analyses were limited to a small number of planned comparisons aligned with these hypotheses, adjustment for multiple testing was not applied. It can be appropriate to assess contrasts when testing specific a priori hypotheses, and it is not necessary to interpret two‐way ANOVA main effects and interaction effects in such cases (Rutherford, 2011; Schad et al., 2020).

A total of 28 participants were included in the final participant‐level analytic dataset; however, complete analyzable pre‐ and post‐infusion data varied by outcome because of technical or procedural limitations. Complete data were available for 10–11 participants per infusion visit for the ICA transient hypercapnia assessment and 17–20 participants per infusion visit for the MCA sustained hypercapnia assessment (Tables S1 and S2). Analyses were conducted using the remaining available data.

The effect of the AA infusion on serum AA was assessed using a Wilcoxon matched‐pairs signed rank test during the AA visit. Given the small sample size, we treated the analyses for the AA visit as exploratory and only present the results descriptively for the SAL visit. Data from uncorrected Fisher's Least Significant Difference tests, Wilcoxon matched‐pairs signed rank test, and unpaired Mann–Whitney tests are presented in graphs as means and individual data points and in tables as mean ± SD. Differences in TCD depth and power were analyzed using mixed‐effect models. Spearman correlations were used to correlate the baseline ICA (transient CVR) and brachial artery (FMD) endothelial responses during the AA infusion visit. GraphPad Prism 10.3.0 was used for all statistical analyses, and statistical significance was set at *p* < 0.05 for all analyses.

## 3 RESULTS

### 3.1 Descriptive characteristics

Group participant characteristics for Y and MA/O adults are presented in Table S3. Both groups were well balanced for sex (Y: 56%/44% and MA/O: 42%/58%, men/women, Table S3). MA/O adults exhibited higher BMI (*p* = 0.0014) and MAP (*p* = 0.038). Systolic blood pressure (*p* = 0.062), diastolic blood pressure (*p* = 0.058), HR (*p* = 0.066), and blood glucose (*p* = 0.058) were higher but not significant in MA/O adults. There were no differences in global cognition or total recall between age groups (Table S3).

### 3.2 The effect of AA on transient ICA CVR

Relative (%) ICA CVR to hypercapnia was significantly lower at baseline (prior to AA infusion) in MA/O adults compared to Y adults and was improved following the infusion of AA (Table 1, Figure 1a). Although absolute ICA CVR at baseline did not differ between age groups, it significantly increased in MA/O adults following AA infusion (Table 1, Figure 1b). No age or infusion effects were observed on absolute or relative ICA CVR during the SAL visit (Table 1, Figure 1c,d). Likewise, there were no effects on the absolute change in heart rate or P_ET_CO_2_ in response to transient hypercapnia (Table 1). MA/O adults had a blunted MAP response to transient hypercapnia, compared to Y adults pre‐AA; however, neither infusion altered the MAP response to transient hypercapnia between Y and MA/O adults (Table 1).

**TABLE 1:** ICA response to transient hypercapnia.

Variable	SAL				AA			
	Y adults		MA/O adults		Y adults		MA/O adults	
	Pre	Post	Pre	Post	Pre	Post	Pre	Post
CVR, %	5.37 ± 1.99	4.74 ± 2.44	3.02 ± 2.39	3.62 ± 2.91	3.39 ± 1.71	4.14 ± 1.21	**0.67 ± 1.73** ^b^	**4.79 ± 2.67** ^a^
CVR, mm	0.28 ± 0.12	0.26 ± 0.15	0.19 ± 0.14	0.29 ± 0.25	0.16 ± 0.083	0.20 ± 0.06	0.056 ± 0.11	**0.32 ± 0.24** ^a^
Velocity, Δcm/s	−0.070 ± 2.51	1.34 ± 2.17	−0.89 ± 4.42	−10.74 ± 31.41	3.52 ± 6.53	5.44 ± 4.27	1.61 ± 2.49	0.95 ± 3.01
Shear rate, Δsec^−1^	−4.41 ± 21.44	−0.33 ± 22.41	−9.27 ± 26.24	30.44 ± 32.01	31.49 ± 56.83	33.42 ± 25.64	13.92 ± 19.23	3.27 ± 20.87
ΔMAP, mmHg	2.5 ± 2.17	1.67 ± 1.51	0.60 ± 1.95	0.80 ± 3.70	2.60 ± 2.51	0.80 ± 2.40	**−1.50 ± 2.65** ^b^	0.00 ± 1.41
ΔHR, bpm	2.33 ± 9.65	1.0 ± 4.72	1.0 ± 1.41	1.0 ± 2.0	6.60 ± 7.40	−0.40 ± 4.51	3.75 ± 3.30	3.0 ± 3.37
ΔP_ET_CO_2_, mmHg	8.18 ± 0.39	8.68 ± 1.39	8.47 ± 1.26	8.84 ± 1.13	8.50 ± 0.18	8.20 ± 0.73	8.86 ± 1.11	9.41 ± 1.49

*Note*: The relative and absolute change in ICA diameter, velocity, and shear rate and the absolute change MAP, HR, and P_ET_CO_2_ in response to transient hypercapnia before and after the infusion of AA or SAL in Y and MA/O adults were assessed using uncorrected Fisher's Least Significant Difference tests. Statistical significance was set at *p* < 0.05 and displayed in bold text.

Abbreviation: AA, ascorbic acid infusion; CVR, cerebrovascular reactivity; HR, heart rate; ICA, internal carotid artery; ICA_V_, ICA velocity; MA/O, middle‐aged and older adults; MAP, mean arterial pressure; P_ET_CO_2_, partial pressure of end‐tidal CO_2_; post, post‐infusion; pre, pre‐infusion; SAL, saline infusion; Y, young adults.

^a^ Pre vs. post‐infusion.

^b^ Pre‐infusion Y vs. MA/O adults within an infusion visit.

**FIGURE 1:**
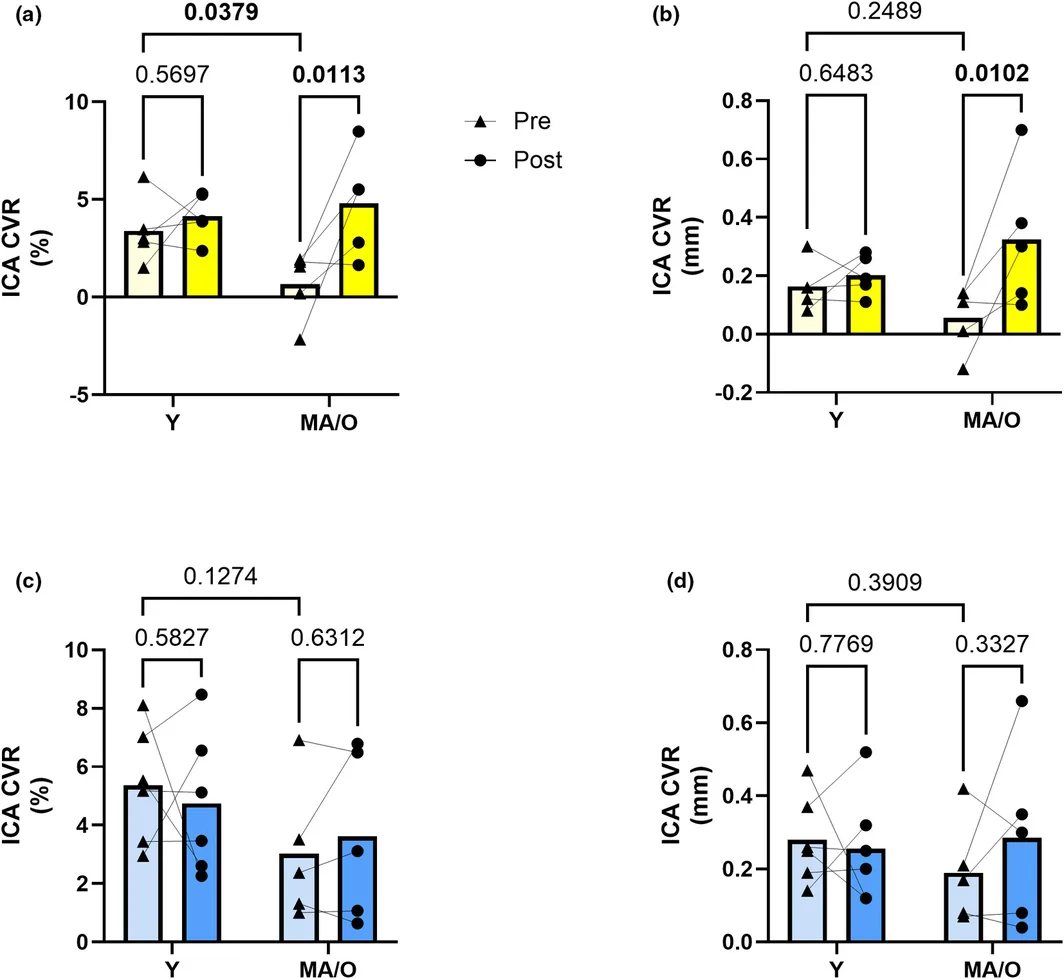
The Effect of AA on ICA CVR. The effect of age (Y vs. MA/O) and infusion (AA vs. SAL) on (a, c) PCT ICA CVR and (b, d) ABS ICA CVR in response to transient hypercapnia between Y and MA/O adults were assessed using uncorrected Fisher's Least Significant Difference tests. Pre‐infusion means are displayed in light color and individual data points as black triangles and post‐infusion means are displayed in dark color and individual data points as circles. AA conditions are shown in yellow and SAL conditions are shown in blue. Statistical significance was set at *p* < 0.05 and significant values are displayed in bold text. Data are presented as means with individual participant data points. AA, ascorbic acid infusion; ABS, absolute; CVR, cerebrovascular reactivity; ICA, internal carotid artery; MA/O, middle‐aged and older adults; PCT, percent; post, post‐infusion; pre, pre‐infusion; SAL, saline infusion; Y, young adults.

### 3.3 The effect of AA on sustained MCA CVR

Relative and absolute MCA CVR were not significantly different at baseline (prior to AA infusion) between Y and MA/O adults when expressed as a change in blood velocity (Table 2, Figure 2a,b). However, MCA CVR was significantly lower in MA/O compared to Y adults when normalized to MAP (MCA_CVC_; Table 2, Figure 2c,d). AA increased MCA CVR in MA/O adults when expressed as a change in velocity or conductance (Table 2, Figure 2a–c). Pre‐SAL infusion percent and absolute MCA_V_ and MCA_CVC_ CVR were not significantly different between Y and MA/O adults (Table 2, Figure 2e–h); however, the SAL infusion increased percent and absolute MCA_V_ CVR in Y adults (Table 2, Figure 2e,f). The augmented CVR in Y adults following SAL infusion could be due to a volume‐loading effect, as the percentage and absolute MCA_CVC_ CVR responses were not significant post‐SAL infusion (Table 2, Figure 2g,h). Further, the absolute change in MAP appeared to be higher following the SAL infusion in Y adults (*p* = 0.063, Table 2), although this difference was not statistically significant. We did not observe an effect of age or SAL infusion on the absolute change in MAP, HR, or P_ET_CO_2_ (Table 2).

**TABLE 2:** MCA response to sustained hypercapnia.

Variable	SAL				AA			
	Y adults		MA/O adults		Y adults		MA/O adults	
	Pre	Post	Pre	Post	Pre	Post	Pre	Post
MCA_V_ CVR, %/mmHg	2.95 ± 0.54	**3.84 ± 1.21** ^a^	3.12 ± 1.11	3.60 ± 1.02	3.39 ± 0.90	3.18 ± 1.05	3.28 ± 1.70	**4.08 ± 1.86** ^a^
MCA_V_ CVR, cm/s/mmHg	1.43 ± 0.38	**2.19 ± 0.89** ^a^	1.75 ± 0.73	2.07 ± 0.72	1.97 ± 0.65	1.92 ± 0.84	1.95 ± 0.79	**2.29 ± 0.39** ^a^
MCA_CVC_ CVR, %/mmHg	1.89 ± 0.56	1.99 ± 0.99	2.13 ± 0.59	2.36 ± 0.94	2.23 ± 4.20	1.71 ± 0.79	**1.32 ± 1.10** ^b^	**2.01 ± 0.53** ^a^
MCA_CVC_ CVR, %/mmHg/mmHg	0.012 ± 0.006	0.014 ± 0.008	0.0013 ± 0.005	0.014 ± 0.006	0.018 ± 0.006	0.014 ± 0.008	**0.009 ± 0.009** ^b^	0.014 ± 0.003
ΔMAP, mmHg	6.19 ± 4.05	10.33 ± 3.64	8.60 ± 2.99	8.77 ± 3.73	6.04 ± 5.85	8.53 ± 1.14	8.68 ± 3.67	9.61 ± 2.96
ΔHR, bpm	5.81 ± 3.91	4.06 ± 5.57	3.75 ± 2.88	4.36 ± 2.39	8.89 ± 8.20	3.53 ± 5.51	3.43 ± 2.85	6.33 ± 8.11
ΔP_ET_CO_2_, mmHg	8.65 ± 0.59	8.39 ± 0.82	8.25 ± 0.89	8.40 ± 0.63	8.55 ± 0.72	7.88 ± 1.23	8.23 ± 0.71	8.37 ± 0.54

*Note*: The relative and absolute change in MCA velocity and conductance and absolute change MAP, HR, and P_ET_CO_2_ in response to sustained hypercapnia before and after the infusion of AA or SAL were assessed in Y and MA/O adults using uncorrected Fisher's Least Significant Difference tests. Statistical significance was set at *p* < 0.05 and displayed in bold text.

Abbreviation: AA, ascorbic acid infusion; CVR, cerebrovascular reactivity; HR, heart rate; MA/O, middle‐aged and older adults; MAP, mean arterial pressure; MCA_CVC_, middle cerebral artery conductance; MCA_V_, middle cerebral artery velocity; P_ET_CO_2_, partial pressure of end‐tidal CO_2_; post, post‐infusion; Pre, pre‐infusion; SAL, saline infusion; Y, young adults.

^a^ Pre vs. post‐infusion.

^b^ Pre‐infusion Y vs. MA/O adults within an infusion visit.

**FIGURE 2:**
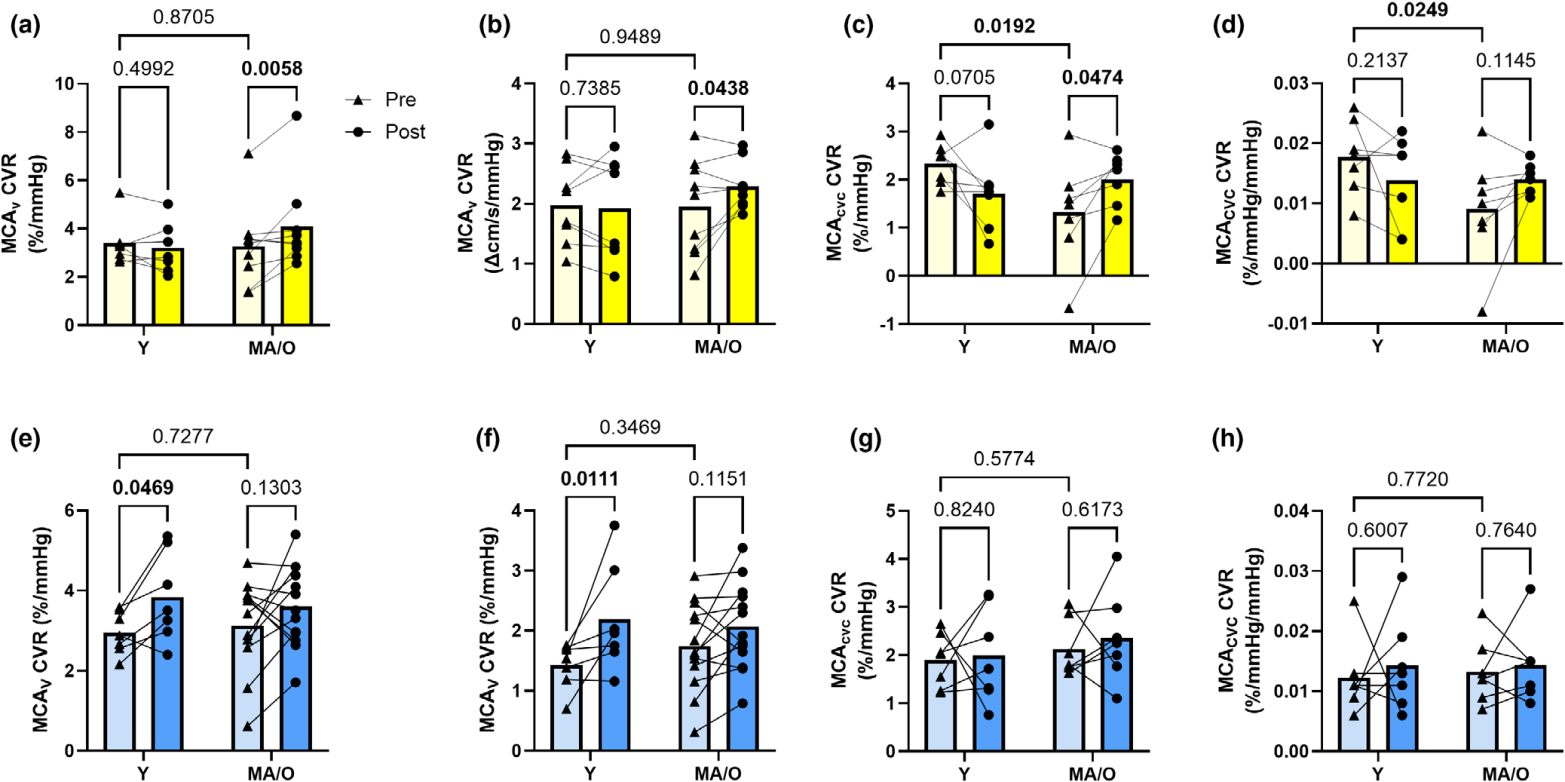
The Effect of AA on MCA CVR. The effect of age (Y vs. MA/O) and infusion (AA vs. SAL) on (a, e) PCT MCA_V_ CVR, (b, f) ABS MCA_V_ CVR, (c, g) PCT MCA_CVC_ CVR, and (d, h) ABS MCA_CVC_ CVR in response to sustained hypercapnia were assessed using uncorrected Fisher's Least Significant Difference tests. Pre‐infusion means are displayed in light color and individual data points as black triangles and post‐infusion means are displayed in dark color and individual data points as circles. AA conditions are shown in yellow and SAL conditions are shown in blue. Statistical significance was set at *p* < 0.05 and significant values are displayed in bold text. Data are presented as means with individual participant data points. AA, ascorbic acid infusion; ABS, absolute; CVR, cerebrovascular reactivity; MA/O, middle‐aged and older adults; MCA_CVC_, MCA cerebrovascular conductance; MCA_V_, middle cerebral artery velocity; PCT, percent; post, post‐infusion; pre, pre‐infusion; SAL, saline infusion; Y, young adults.

### 3.4 The effect of AA on TCBF

Pre‐AA infusion TCBF was significantly higher in MA/O adults compared to Y adults (Table S4), but there was no effect of AA on TCBF in Y or MA/O adults (Table S4). The difference in pre‐AA TCBF may be driven by higher ICA flow in MA/O adults (*p* = 0.066, Table S4); however, this difference only approached statistical significance. There was no effect of age or AA infusion on ICA diameter or VA flow or diameter (Table S4). The SAL infusion appeared to increase TCBF in MA/O adults (*p* = 0.077, Table S4), but this effect was also not statistically significant. Blood pressure was not measured during the TCBF measurement, so we cannot assess whether this was due to a volume‐loading effect. The SAL infusion did not alter ICA or VA flow or diameter, but pre‐SAL ICA diameter was higher in MA/O adults compared to Y adults (Table S4).

### 3.5 The effect of AA on FMD

We observed age‐related differences in FMD, as MA/O adults had significantly lower pre‐AA infusion relative FMD and relative and absolute FMD normalized for shear rate (Table S5). We also observed that pre‐SAL relative and absolute FMD was lower in MA/O adults compared to Y adults (Table S5). While AA infusion appeared to increase relative FMD (*p* = 0.055) in MA/O adults, the effect was not statistically significant (Table S5). There was no effect of age or infusion on any other FMD variable assessed (Table S5). In a small analytic subset with paired data, relative FMD was also not correlated with relative ICA response to transient hypercapnia (Spearman R = 0.11, *p* = 0.84, *n* = 7).

### 3.6 The effect of AA on serum AA

In a limited analytic subset of participants who underwent the same infusion protocol, AA infusion increased serum concentrations of AA by approximately 7‐fold on average in Y and MA/O adults (Pre: 1.19 ± 0.64 vs. Post: 8.64 ± 2.93 mg/dL; Figure S3A,B). These data support that the AA‐infusion protocol may have increased serum AA concentrations in our study.

## 4 DISCUSSION

In this study, the acute infusion of AA augmented cerebrovascular function in MA/O adults, but not Y adults. This finding was demonstrated using ICA CVR during transient hypercapnia and MCA CVR during sustained hypercapnia. These data support the hypothesis that aging may influence extracranial and intracranial cerebrovascular function through redox or antioxidant‐responsive pathways, and that an AA infusion may be an appropriate model to study the age‐related changes in cerebrovascular function.

The transient increase in the partial pressure of arterial carbon dioxide causes a dilation of vessels downstream of the ICA, resulting in an increase in turbulent blood flow and shear stress in the ICA (Hoiland et al., 2017). This shear stress is believed to induce dilation of the ICA through the release of NO from the endothelium (Hoiland et al., 2017). Importantly, NO has recently been confirmed to be a primary mechanism contributing to the ICA shear‐mediated dilatory response to transient hypercapnia, but was not found to contribute to the ICA or MCA response to sustained hypercapnia response (Hoiland et al., 2022). Therefore, ICA CVR appears to be an appropriate assessment of extracranial cerebrovascular NO‐mediated endothelial function. ICA CVR in response to transient hypercapnia has previously been shown to be lower in older adults compared to younger adults (Freeberg et al., 2025; Iwamoto et al., 2018), an observation which our study confirmed. Our finding that AA increased ICA CVR in MA/O adults is consistent with the broader hypothesis that age‐related cerebrovascular dysfunction may be influenced by redox‐related mechanisms. However, because oxidative stress and NO bioavailability were not directly measured, these mechanisms warrant further investigation.

MCA CVR, on the other hand, is a less NO‐dependent assessment of intracranial cerebrovascular function (Carr et al., 2021; Hoiland et al., 2019, 2022). NO appears to only play a permissive role to MCA CVR in response to sustained hypercapnia. Thus, the mechanism through which AA increased ICA CVR in MA/O adults seems more plausibly dependent on NO than the mechanism through which it increased MCA CVR. The mechanisms underlying the MCA CVR response are complex and not fully understood, but may include pH balance, red blood cell ATP release, adenosine, cyclic GMP, K^+^ and Ca^2+^ channels, reactive oxygen species‐induced vasodilation (Rosenblum, 1983) and prostaglandin and cyclooxygenase activity (Carr et al., 2021; Hoiland et al., 2019). Therefore, additional research is needed to elucidate the mechanism through which aging blunts MCA CVR in MA/O adults.

Our finding that AA increased MCA CVR is contrary to a recent study which observed that AA did not alter MCA CVR in older adults (Freeberg et al., 2025). However, sex differences were observed in the prior study, where AA restored MCA CVR in older women (Freeberg et al., 2025). Differences in hypercapnia stimulus and delivery and lack of baseline measurement in the prior study may explain the discordant findings. While we observed that SAL augmented MCA CVR in Y adults, this effect was abolished after controlling for MAP (MCA_CVC_). Further, the absolute change in MAP during sustained hypercapnia appeared higher in Y adults following the SAL infusion. These findings may indicate that the augmented MCA CVR following SAL infusion was due to volume loading, rather than altered cerebrovascular function.

We did not observe a correlation between cerebrovascular and peripheral vascular endothelial function in our study in Y and MA/O adults. This observation is consistent with and extends findings from a prior study in young adults–which the authors discuss the lack of correlation could be explained by differences in baseline diameter and shear‐rate caused by differences in innervation, arterial structure and downstream resistance, paracrine signaling, and endothelial phenotype (Carr et al., 2020). These results suggest that ICA and brachial artery endothelial responses may reflect distinct endothelial physiology and should not be interpreted as interchangeable measures of endothelial function. The inconsistent effect of age on measures of cerebrovascular and peripheral function in this study may also reflect differences in sample size across time points and visits and measurement variability. Despite these limitations, we observed that pre‐infusion percentage FMD was significantly lower in MA/O adults compared to Y during each study visit. Further, percent CVR in the ICA was significantly lower in MA/O adults compared to Y adults at baseline, prior to the AA‐infusion, as was the MCA, after adjusting for MAP. Collectively, these findings suggest at least some age‐related reduction in peripheral and cerebrovascular endothelial function and intracranial cerebrovascular function, despite inconsistencies across all analyses.

Unexpectedly, TCBF was significantly higher in MA/O adults than in Y adults before AA infusion, potentially reflecting baseline differences in ICA blood flow and/or blood pressure. This is contrary to prior literature in which age‐related differences in cerebral blood flow were observed (Albayrak et al., 2007; Buijs et al., 1998; Catchlove et al., 2018; Kawano et al., 2024; Liu et al., 2012; Lu et al., 2011; Parkes et al., 2004; Scheel, Ruge, Petruch, & Schöning, 2000; Scheel, Ruge, & Schöning, 2000; Stoquart‐ElSankari et al., 2007; Tarumi et al., 2014; Tomoto et al., 2023; Yazici et al., 2005; Zhang et al., 2018). Because TCBF provides a global estimate of cerebral blood flow, it cannot capture potential regional differences in age‐related blood flow patterns across different regions and vessels in the brain (Alisch et al., 2021; Kawano et al., 2024; Liu et al., 2012; Lu et al., 2011; Parkes et al., 2004; Zarrinkoob et al., 2015; Zhang et al., 2018). Thus, although global blood flow was higher in MA/O adults, we could not determine whether regional cerebral perfusion differed between groups. Biological sex may also influence the age‐related difference in TCBF, as women have been found to have higher TCBF compared to men, and several of these studies observed this sex difference to be consistent, regardless of age (Alisch et al., 2021; Kawano et al., 2024; Liu et al., 2012; Lu et al., 2011; Parkes et al., 2004; Xing et al., 2017; Zhang et al., 2018). In our study, the proportion of women in the subset with available TCBF data was greater in our MA/O group (71%) compared to the Y group (44%), which may explain our observation that MA/O adults had higher TCBF. Additional research is needed to examine the effect of oxidative stress on TCBF in men and women, as oxidative stress related to estrogen loss may alter cerebrovascular function in women. For instance, findings from a murine study show that oxidative stress may augment cerebral blood flow through a nicotinamide adenine dinucleotide phosphate oxidase‐dependent mechanism caused by loss of estrogen (Miller et al., 2007). Converging observational research supports this finding, as oxidative stress was higher in post‐menopausal women compared to age‐matched men (Bourgonje et al., 2020; Cakir et al., 2016; Vassalle et al., 2012). Overall, the higher TCBF observed in MA/O adults in our study should be interpreted cautiously and may reflect the composition of the analytic sample, measurement variability, or hemodynamic conditions during assessment, rather than a generalized age‐related increase in cerebral perfusion.

Our study has several limitations. First, our sample size was relatively small, varied across measurements, and was insufficient to assess sex or racial/ethnic differences. Second, our assessment of MCA CVR using TCD assumes that the MCA does not dilate during hypercapnia, and this assumption has been challenged by data from multiple studies assessing MCA diameter using MRI (Al‐Khazraji et al., 2021; Coverdale et al., 2014, 2015; Miller et al., 2019; Serrador et al., 2000; Verbree et al., 2014). However, MCA dilation during hypercapnia may be influenced by other factors such as the CVR protocol and aging (Al‐Khazraji et al., 2021; Miller et al., 2019; Serrador et al., 2000; Verbree et al., 2014); suggesting that measuring MCA_V_ during sustained hypercapnia may be appropriate in certain contexts. Third, the assessment of ICA vasodilation is very technically challenging and would be difficult to implement clinically. Fourth, because we did not include a comparable vasodilatory control condition, we cannot determine whether the AA effect was specific to NO‐dependent vasodilation. Fifth, substantial inter‐individual variability was observed in several cerebrovascular outcomes, including ICA CVR. In addition, age‐related differences were not consistently present at baseline across the AA and SAL visits. These findings could reflect biological heterogeneity, inter‐day variability, measurement variability, or the limited sample size. Although the randomized crossover design was intended to minimize potential order effects, we cannot completely exclude their contribution to the observed variability. Future studies with larger sample sizes and repeated assessments are needed to better characterize the reproducibility and determinants of cerebrovascular responses to hypercapnia. Sixth, we did not measure beat‐to‐beat blood pressure during the TCBF assessment; thus, we are unable to interpret changes in cerebral blood flow independent of perfusion pressure. Seventh, because of a freezer malfunction, we were unable to measure serum AA, oxidative stress markers, or NO‐related biomarkers in the full cohort. Consequently, we chose to focus the biomarker analysis on the measurement of AA in a limited analytic subset of participants. This exploratory analysis should be interpreted with caution given the small sample size and limited statistical power. Lastly, it is difficult to discern with the present data set whether the augmented ICA and MCA CVR in MA/O adults were due to reductions in oxidative stress, increases in NO, or AA acting through entirely separate mechanisms.

In summary, we found that age‐related differences in CVR were detected in the ICA and MCA in response to transient and sustained hypercapnia, respectively. AA increased ICA and MCA CVR in MA/O adults, whereas similar increases were not observed during the SAL visit. Collectively, these findings suggest that antioxidant‐responsive mechanisms may be implicated in the regulation of age‐related impairments in extracranial and intracranial cerebrovascular reactivity, supporting further investigation of redox pathways as potential targets for preserving cerebrovascular function with aging.

## AUTHOR CONTRIBUTIONS

**Theodore M. DeConne:** Data curation; formal analysis; project administration. **Faria Sanjana:** Data curation. **Elizabeth M. Habash:** Data curation; formal analysis; project administration. **Kevin P. Decker:** Data curation; formal analysis. **Wendy Nichols:** Data curation. **Joshua C. Hobson:** Project administration. **Nicholas A. Rizzi:** Data curation; formal analysis. **Christopher R. Martens:** Conceptualization; funding acquisition; methodology; project administration; supervision.

## FUNDING INFORMATION

This research was funded by P20 GM113125 and K01 AG054731.

## CONFLICT OF INTEREST STATEMENT

The authors declare no conflicts of interest.

## Supporting information


**Table S1.** File‐level analytic counts for transient hypercapnia stimulus.
**Table S2.** File‐level analytic counts for sustained hypercapnia stimulus.
**Table S3.** Participant‐level final characteristics.
**Table S4.** AA did not alter TCBF in Y or MA/O adults.
**Table S5.** AA did not alter FMD in Y or MA/O adults.
**Figure S1.** Protocol figure. Y (18–29 years) and MA/O (55–79 years) community‐dwelling adults were enrolled in a placebo‐controlled, randomized, crossover, intervention study. SAL (blue) and AA (yellow) infusions were used to “pharmaco‐dissect” the effect of aging on cerebrovascular and peripheral vascular function. A minimum 4‐day washout separated each infusion visit. FMD, TCBF, ICA CVR in response to transient hypercapnia, and MCA CVR in response to sustained hypercapnia were assessed before and after each infusion. Participants received a bolus infusion followed by a drip infusion that was sustained until all post‐infusion vascular measurements were made. Abbreviations: Y, young; MA/O, middle‐aged and older; SAL, saline infusion; AA, ascorbic acid infusion; FMD, flow mediated dilation; TCBF, total cerebral blood flow; MCA, middle cerebral artery; CVR, cerebrovascular reactivity; and ICA, internal carotid artery.
**Figure S2.** Differences between transcranial Doppler depth and power. The transcranial Doppler (A) depth and (B) power used to insonate the middle cerebral artery were assessed before and after each infusion using Mixed‐effects models. Data are presented as mean ± SD. Abbreviations: TCD, transcranial Doppler; SAL, saline; and AA, ascorbic acid.
**Figure S3.** The effect of AA infusion on serum concentrations. Serum AA was assessed in (A) Y and MA/O adults before and after the infusion of AA and in (B) MA/O adults before and after the infusion of SAL. Serum AA measured during the SAL visit is only presented descriptively, given the small sample size. Pre‐infusion means are displayed in light color and post‐infusion means are displayed in dark color. Individual data points from Y and MA/O adults are displayed as black triangles and circles, respectively. Data are presented as mean ± SD. Abbreviations: SAL, saline; AA, ascorbic acid; Y, young adults; and MA/O, middle‐aged and older.

## Data Availability

Data will be made available upon reasonable request. Please contact the corresponding author to request the raw data.

## References

[phy271120-bib-0001] Albayrak, R. , Degırmencı, B. , Acar, M. , Haktanır, A. , Colbay, M. , & Yaman, M. (2007). Doppler sonography evaluation of flow velocity and volume of the extracranial internal carotid and vertebral arteries in healthy adults. Journal of Clinical Ultrasound, 35, 27–33. 10.1002/jcu.20301 17149761

[phy271120-bib-0002] Alisch, J. S. R. , Khattar, N. , Kim, R. W. , Cortina, L. E. , Rejimon, A. C. , Qian, W. , Ferrucci, L. , Resnick, S. M. , Spencer, R. G. , & Bouhrara, M. (2021). Sex and age‐related differences in cerebral blood flow investigated using pseudo‐continuous arterial spin labeling magnetic resonance imaging. Aging, 13, 4911–4925. 10.18632/aging.202673 PMC7950235 33596183

[phy271120-bib-0003] Al‐Khazraji, B. K. , Buch, S. , Kadem, M. , Matushewski, B. J. , Norozi, K. , Menon, R. S. , & Shoemaker, J. K. (2021). Protocol‐dependence of middle cerebral artery dilation to modest hypercapnia. Applied Physiology, Nutrition, and Metabolism, 46, 1038–1046. 10.1139/apnm-2021-0220 34139129

[phy271120-bib-0004] Alwatban, M. , Murman, D. L. , & Bashford, G. (2019). Cerebrovascular reactivity impairment in preclinical Alzheimer's disease. Journal of Neuroimaging, 29, 493–498. 10.1111/jon.12606 30748053

[phy271120-bib-0005] Aslanyan, V. , Mack, W. J. , Ortega, N. E. , Nasrallah, I. M. , Pajewski, N. M. , Williamson, J. D. , & Pa, J. (2024). Cerebrovascular reactivity in Alzheimer's disease signature regions is associated with mild cognitive impairment in adults with hypertension. Alzheimer's and Dementia, 20, 1784–1796. 10.1002/alz.13572 PMC10984494 38108158

[phy271120-bib-0006] Bourgonje, A. R. , Abdulle, A. E. , Al‐Rawas, A. M. , Al‐Maqbali, M. , Al‐Saleh, M. , Enriquez, M. B. , Al‐Siyabi, S. , Al‐Hashmi, K. , Al‐Lawati, I. , Bulthuis, M. L. C. , Mulder, D. J. , Gordijn, S. J. , Van Goor, H. , & Saleh, J. (2020). Systemic oxidative stress is increased in postmenopausal women and independently associates with homocysteine levels. International Journal of Molecular Sciences, 21, 314. 10.3390/ijms21010314 PMC6982320 31906485

[phy271120-bib-0007] Buijs, P. C. , Krabbe‐Hartkamp, M. J. , Bakker, C. J. , de Lange, E. E. , Ramos, L. M. , Breteler, M. M. , & Mali, W. P. (1998). Effect of age on cerebral blood flow: Measurement with ungated two‐dimensional phase‐contrast MR angiography in 250 adults. Radiology, 209, 667–674. 10.1148/radiology.209.3.9844657 9844657

[phy271120-bib-0008] Cakir, T. , Goktas, B. , Mutlu, M. F. , Mutlu, I. , Bilgihan, A. , Erdem, M. , & Erdem, A. (2016). Advanced oxidation protein products and malondialdehyde — The new biological markers of oxidative stress — Are elevated in postmenopausal women. Ginekologia Polska, 87, 321–325. 10.5603/GP.2016.0001 27304645

[phy271120-bib-0009] Carr, J. M. J. R. , Caldwell, H. G. , & Ainslie, P. N. (2021). Cerebral blood flow, cerebrovascular reactivity and their influence on ventilatory sensitivity. Experimental Physiology, 106, 1425–1448. 10.1113/EP089446 33932955

[phy271120-bib-0010] Carr, J. M. J. R. , Hoiland, R. L. , Caldwell, H. G. , Coombs, G. B. , Howe, C. A. , Tremblay, J. C. , Green, D. J. , & Ainslie, P. N. (2020). Internal carotid and brachial artery shear‐dependent vasodilator function in young healthy humans. The Journal of Physiology, 598, 5333–5350. 10.1113/JP280369 32901919

[phy271120-bib-0011] Catchlove, S. J. , Macpherson, H. , Hughes, M. E. , Chen, Y. , Parrish, T. B. , & Pipingas, A. (2018). An investigation of cerebral oxygen utilization, blood flow and cognition in healthy aging. PLoS One, 13, 1–21. 10.1371/journal.pone.0197055 PMC5963791 29787609

[phy271120-bib-0012] Chen, P. , Reed, G. , Jiang, J. , Wang, Y. , Sunega, J. , Dong, R. , Ma, Y. , Esparham, A. , Ferrell, R. , Levine, M. , Drisko, J. , & Chen, Q. (2022). Pharmacokinetic evaluation of intravenous vitamin C: A classic pharmacokinetic study. Clinical Pharmacokinetics, 61, 1237–1249. 10.1007/s40262-022-01142-1 PMC9439974 35750958

[phy271120-bib-0013] Coverdale, N. S. , Gati, J. S. , Opalevych, O. , Perrotta, A. , & Shoemaker, J. K. (2014). Cerebral blood flow velocity underestimates cerebral blood flow during modest hypercapnia and hypocapnia. Journal of Applied Physiology, 117, 1090–1096. 10.1152/japplphysiol.00285.2014 25012027

[phy271120-bib-0014] Coverdale, N. S. , Lalande, S. , Perrotta, A. , & Shoemaker, J. K. (2015). Heterogeneous patterns of vasoreactivity in the middle cerebral and internal carotid arteries. American Journal of Physiology. Heart and Circulatory Physiology, 308, H1030–H1038. 10.1152/ajpheart.00761.2014 25724496

[phy271120-bib-0015] Delis, D. C. , Kramer, J. H. , Kaplan, E. , & Ober, B. A. (2022). California Verbal Learning Test, Third Edition.

[phy271120-bib-0016] Duan, W. , Zhou, G. D. , Balachandrasekaran, A. , Bhumkar, A. B. , Boraste, P. B. , Becker, J. T. , Kuller, L. H. , Lopez, O. L. , Gach, H. M. , & Dai, W. (2021). Cerebral blood flow predicts conversion of mild cognitive impairment into Alzheimer's disease and cognitive decline: An arterial spin labeling follow‐up study. Journal of Alzheimer's Disease, 82, 293–305. 10.3233/JAD-210199 PMC8527573 34024834

[phy271120-bib-0017] Edwards, M. R. , Devitt, D. L. , & Hughson, R. L. (2004). Two‐breath CO _2_ test detects altered dynamic cerebrovascular autoregulation and CO _2_ responsiveness with changes in arterial Pco_2_ . American Journal of Physiology ‐ Regulatory, Integrative and Comparative Physiology, 287, R627–R632. 10.1152/ajpregu.00384.2003 15044183

[phy271120-bib-0018] Eskurza, I. , Monahan, K. D. , Robinson, J. A. , & Seals, D. R. (2004). Effect of acute and chronic ascorbic acid on flow‐mediated dilatation with sedentary and physically active human ageing. The Journal of Physiology, 556, 315–324. 10.1113/jphysiol.2003.057042 PMC1664895 14754992

[phy271120-bib-0019] Freeberg, K. A. , McCarty, N. P. , Chonchol, M. , Seals, D. R. , & Craighead, D. H. (2025). Oxidative stress suppresses internal carotid artery dilation to hypercapnia in healthy older adults. Journal of Applied Physiology (1985), 138, 536–545. 10.1152/japplphysiol.00322.2024 PMC12167614 39819040

[phy271120-bib-0020] Higashi, Y. , Kihara, Y. , & Noma, K. (2012). Endothelial dysfunction and hypertension in aging. Hypertension Research, 35, 1039–1047. 10.1038/hr.2012.138 22972557

[phy271120-bib-0021] Hoiland, R. L. , Caldwell, H. G. , Howe, C. A. , Stacey, B. S. , Dawkins, T. , Wakeham, D. J. , Tremblay, J. C. , Tymko, M. M. , Patrician, A. , Smith, K. J. , Sekhon, M. S. , MacLeod, D. B. , Green, D. J. , Bailey, D. M. , & Ainslie, P. N. (2022). Nitric oxide contributes to cerebrovascular shear‐mediated dilatation but not steady‐state cerebrovascular reactivity to carbon dioxide. The Journal of Physiology, 600, 1385–1403. 10.1113/JP282427 34904229

[phy271120-bib-0022] Hoiland, R. L. , Fisher, J. A. , & Ainslie, P. N. (2019). Regulation of the cerebral circulation by arterial carbon dioxide. Comprehensive Physiology, 9, 1101–1154. 10.1002/j.2040-4603.2019.tb00083.x 31187899

[phy271120-bib-0023] Hoiland, R. L. , Smith, K. J. , Carter, H. H. , Lewis, N. C. S. , Tymko, M. M. , Wildfong, K. W. , Bain, A. R. , Green, D. J. , & Ainslie, P. N. (2017). Shear‐mediated dilation of the internal carotid artery occurs independent of hypercapnia. American Journal of Physiology. Heart and Circulatory Physiology, 313, H24–H31. 10.1152/ajpheart.00119.2017 28389602

[phy271120-bib-0024] Iwamoto, E. , Bock, J. M. , & Casey, D. P. (2018). Hypercapnia‐induced shear‐mediated dilation in the internal carotid artery is blunted in healthy older adults. American Journal of Physiology. Heart and Circulatory Physiology, 315, H1279–H1286. 10.1152/ajpheart.00335.2018 30118339

[phy271120-bib-0025] Jablonski, K. L. , Racine, M. L. , Geolfos, C. J. , Gates, P. E. , Chonchol, M. , McQueen, M. B. , & Seals, D. R. (2013). Dietary sodium restriction reverses vascular endothelial dysfunction in middle‐aged/older adults with moderately elevated systolic blood pressure. Journal of the American College of Cardiology, 61, 335–343. 10.1016/j.jacc.2012.09.010 PMC3549053 23141486

[phy271120-bib-0026] Jablonski, K. L. , Seals, D. R. , Eskurza, I. , Monahan, K. D. , & Donato, A. J. (2007). High‐dose ascorbic acid infusion abolishes chronic vasoconstriction and restores resting leg blood flow in healthy older men. Journal of Applied Physiology, 103, 1715–1721. 10.1152/japplphysiol.00533.2007 17965239

[phy271120-bib-0027] Kawano, H. , Yamada, S. , Watanabe, Y. , Ii, S. , Otani, T. , Ito, H. , Okada, K. , Iseki, C. , Tanikawa, M. , Wada, S. , Oshima, M. , Mase, M. , & Yoshida, K. (2024). Aging and sex differences in brain volume and cerebral blood flow. Aging and Disease, 15, 2216–2229. 10.14336/AD.2023.1122 PMC11346398 38029394

[phy271120-bib-0028] Liu, Y. , Zhu, X. , Feinberg, D. , Guenther, M. , Gregori, J. , Weiner, M. W. , & Schuff, N. (2012). Arterial spin labeling MRI study of age and gender effects on brain perfusion hemodynamics. Magnetic Resonance in Medicine, 68, 912–922. 10.1002/mrm.23286 22139957

[phy271120-bib-0029] Lu, H. , Xu, F. , Rodrigue, K. M. , Kennedy, K. M. , Cheng, Y. , Flicker, B. , Hebrank, A. C. , Uh, J. , & Park, D. C. (2011). Alterations in cerebral metabolic rate and blood supply across the adult lifespan. Cerebral Cortex, 21, 1426–1434. 10.1093/cercor/bhq224 PMC3097991 21051551

[phy271120-bib-0030] Martens, C. R. , & Seals, D. R. (2016). Practical alternatives to chronic caloric restriction for optimizing vascular function with ageing. Journal of Physiology, 594, 7177–7195. 10.1113/JP272348 PMC5157076 27641062

[phy271120-bib-0031] Miller, A. A. , Drummond, G. R. , Mast, A. E. , Schmidt, H. H. H. W. , & Sobey, C. G. (2007). Effect of gender on NADPH‐oxidase activity, expression, and function in the cerebral circulation: Role of estrogen. Stroke, 38, 2142–2149. 10.1161/STROKEAHA.106.477406 17525399

[phy271120-bib-0032] Miller, K. B. , Howery, A. J. , Rivera‐Rivera, L. A. , Johnson, S. C. , Rowley, H. A. , Wieben, O. , & Barnes, J. N. (2019). Age‐related reductions in cerebrovascular reactivity using 4D flow MRI. Frontiers in Aging Neuroscience, 11, 281. 10.3389/fnagi.2019.00281 PMC6811507 31680935

[phy271120-bib-0033] Moreau, K. L. , Gavin, K. M. , Plum, A. E. , & Seals, D. R. (2005). Oxidative stress contributes to chronic leg vasoconstriction in estrogen‐deficient postmenopausal women. Hypertension, 45, 1107–1112. 10.1161/01.HYP.0000165678.63373.8c 17110511

[phy271120-bib-0034] Moreau, K. L. , Stauffer, B. L. , Kohrt, W. M. , & Seals, D. R. (2013). Essential role of estrogen for improvements in vascular endothelial function with endurance exercise in postmenopausal women. The Journal of Clinical Endocrinology and Metabolism, 98, 4507–4515. 10.1210/jc.2013-2183 PMC3816259 24092827

[phy271120-bib-0035] Mutlu‐Türkoğlu, Ü. , İlhan, E. , Öztezcan, S. , Kuru, A. , Aykaç‐Toker, G. , & Uysal, M. (2003). Age‐related increases in plasma malondialdehyde and protein carbonyl levels and lymphocyte DNA damage in elderly subjects. Clinical Biochemistry, 36, 397–400. 10.1016/S0009-9120(03)00035-3 12849873

[phy271120-bib-0036] Padayatty, S. J. , Sun, H. , Wang, Y. , Riordan, H. D. , Hewitt, S. M. , Katz, A. , Wesley, R. A. , & Levine, M. (2004). Vitamin C pharmacokinetics: Implications for Oral and intravenous use. Annals of Internal Medicine, 140, 533–537. 10.7326/0003-4819-140-7-200404060-00010 15068981

[phy271120-bib-0037] Parkes, L. M. , Rashid, W. , Chard, D. T. , & Tofts, P. S. (2004). Normal cerebral perfusion measurements using arterial spin labeling: Reproducibility, stability, and age and gender effects. Magnetic Resonance in Medicine, 51, 736–743. 10.1002/mrm.20023 15065246

[phy271120-bib-0038] Peng, S.‐L. L. , Chen, X. , Li, Y. , Rodrigue, K. M. , Park, D. C. , & Lu, H. (2018). Age‐related changes in cerebrovascular reactivity and their relationship to cognition: A four‐year longitudinal study. NeuroImage, 174, 257–262. 10.1016/j.neuroimage.2018.03.033 PMC5949266 29567504

[phy271120-bib-0039] Pierce, G. L. , Lesniewski, L. A. , Lawson, B. R. , Beske, S. D. , & Seals, D. R. (2009). Nuclear factor‐kappa B activation contributes to vascular endothelial dysfunction via oxidative stress in overweight/obese middle‐aged and older humans. Circulation, 119, 1284–1292. 10.1161/CIRCULATIONAHA.108.804294 PMC2810548 19237660

[phy271120-bib-0040] Pinchuk, I. , Weber, D. , Kochlik, B. , Stuetz, W. , Toussaint, O. , Debacq‐Chainiaux, F. , Dollé, M. E. T. , Jansen, E. H. J. M. , Gonos, E. S. , Sikora, E. , Breusing, N. , Gradinaru, D. , Sindlinger, T. , Moreno‐Villanueva, M. , Bürkle, A. , Grune, T. , & Lichtenberg, D. (2019). Gender‐ and age‐dependencies of oxidative stress, as detected based on the steady state concentrations of different biomarkers in the MARK‐AGE study. Redox Biology, 24, 101204. 10.1016/j.redox.2019.101204 PMC6477672 31022674

[phy271120-bib-0041] Rosenblum, W. I. (1983). Effects of free radical generation on mouse pial arterioles: Probable role of hydroxyl radicals. American Journal of Physiology. Heart and Circulatory Physiology, 245, H139–H142. 10.1152/ajpheart.1983.245.1.H139 6307066

[phy271120-bib-0042] Rossman, M. J. , Santos‐Parker, J. R. , Steward, C. A. C. C. , Bispham, N. Z. , Cuevas, L. M. , Rosenberg, H. L. , Woodward, K. A. , Chonchol, M. , Gioscia‐Ryan, R. A. , Murphy, M. P. , & Seals, D. R. (2018). Chronic supplementation with a mitochondrial antioxidant (MitoQ) improves vascular function in healthy older adults. Hypertension, 71, 10787. 10.1161/HYPERTENSIONAHA.117.10787 PMC5945293 29661838

[phy271120-bib-0043] Rutherford, A. (2011). ANOVA and ANCOVA: A GLM Approach (2nd ed.). Wiley.

[phy271120-bib-0044] Schad, D. J. , Vasishth, S. , Hohenstein, S. , & Kliegl, R. (2020). How to capitalize on a priori contrasts in linear (mixed) models: A tutorial. Journal of Memory and Language, 110, 104038. 10.1016/j.jml.2019.104038

[phy271120-bib-0045] Scheel, P. , Ruge, C. , Petruch, U. R. , & Schöning, M. (2000). Color duplex measurement of cerebral blood flow volume in healthy adults. Stroke, 31, 147–150. 10.1161/01.STR.31.1.147 10625730

[phy271120-bib-0046] Scheel, P. , Ruge, C. , & Schöning, M. (2000). Flow velocity and flow volume measurements in the extracranial carotid and vertebral arteries in healthy adults: Reference data and the effects of age. Ultrasound in Medicine & Biology, 26, 1261–1266. 10.1016/S0301-5629(00)00293-3 11120363

[phy271120-bib-0047] Seals, D. R. R. , Jablonski, K. L. L. , & Donato, A. J. J. (2011). Aging and vascular endothelial function in humans. Clinical Science, 120, 357–375. 10.1042/CS20100476 PMC3482987 21244363

[phy271120-bib-0048] Serrador, J. M. , Picot, P. A. , Rutt, B. K. , Shoemaker, J. K. , & Bondar, R. L. (2000). MRI measures of middle cerebral artery diameter in conscious humans during simulated orthostasis. Stroke, 31, 1672–1678. 10.1161/01.STR.31.7.1672 10884472

[phy271120-bib-0049] Sies, H. (1997). Oxidative stress: Oxidants and antioxidants. Experimental Physiology, 82, 291–295. 10.1113/expphysiol.1997.sp004024 9129943

[phy271120-bib-0050] Smalheiser, N. R. (2017). Nonparametric tests. In Data Literacy (pp. 157–167). Elsevier.

[phy271120-bib-0051] Stoquart‐ElSankari, S. , Balédent, O. , Gondry‐Jouet, C. , Makki, M. , Godefroy, O. , & Meyer, M.‐E. (2007). Aging effects on cerebral blood and cerebrospinal fluid flows. Journal of Cerebral Blood Flow and Metabolism, 27, 1563–1572. 10.1038/sj.jcbfm.9600462 17311079

[phy271120-bib-0052] Sur, S. , Lin, Z. , Li, Y. , Yasar, S. , Rosenberg, P. , Moghekar, A. , Hou, X. , Kalyani, R. , Hazel, K. , Pottanat, G. , Xu, C. , van Zijl, P. , Pillai, J. , Liu, P. , Albert, M. , & Lu, H. (2020). Association of cerebrovascular reactivity and Alzheimer pathologic markers with cognitive performance. Neurology, 95, e962–e972. 10.1212/WNL.0000000000010133 PMC7668551 32661101

[phy271120-bib-0053] Tan, B. L. , Norhaizan, M. E. , Liew, W.‐P.‐P. , & Sulaiman Rahman, H. (2018). Antioxidant and oxidative stress: A mutual interplay in age‐related diseases. Frontiers in Pharmacology, 9, 1–2. 10.3389/fphar.2018.01162 PMC6204759 30405405

[phy271120-bib-0054] Tarumi, T. , Ayaz Khan, M. , Liu, J. , Tseng, B. Y. , Parker, R. , Riley, J. , Tinajero, C. , & Zhang, R. (2014). Cerebral hemodynamics in normal aging: Central artery stiffness, wave reflection, and pressure pulsatility. Journal of Cerebral Blood Flow and Metabolism, 34, 971–978. 10.1038/jcbfm.2014.44 PMC4050241 24643081

[phy271120-bib-0055] Thijssen, D. H. J. , Black, M. A. , Pyke, K. E. , Padilla, J. , Atkinson, G. , Harris, R. A. , Parker, B. , Widlansky, M. E. , Tschakovsky, M. E. , & Green, D. J. (2011). Assessment of flow‐mediated dilation in humans: A methodological and physiological guideline. American Journal of Physiology Heart and Circulatory Physiology, 300, H2–H12. 10.1152/ajpheart.00471.2010 PMC3023245 20952670

[phy271120-bib-0056] Tomoto, T. , Lu, M. , Khan, A. M. , Liu, J. , Pasha, E. P. , Tarumi, T. , & Zhang, R. (2023). Cerebral blood flow and cerebrovascular resistance across the adult lifespan: A multimodality approach. Journal of Cerebral Blood Flow and Metabolism, 43, 962–976. 10.1177/0271678X231153741 PMC10196748 36708213

[phy271120-bib-0057] Ungvari, Z. , Kaley, G. , Cabo, R. D. , Sonntag, W. E. , & Csiszar, A. (2018). Mechanisms of vascular aging : New Perspectives. The Journals of Gerontology. Series A, Biological Sciences and Medical Sciences, 65, 1028–1041. 10.1093/gerona/glq113 PMC2950814 20576649

[phy271120-bib-0058] Ungvari, Z. , Tarantini, S. , Kiss, T. , Wren, J. D. , Giles, C. B. , Griffin, C. T. , Murfee, W. L. , Pacher, P. , & Csiszar, A. (2018). Endothelial dysfunction and angiogenesis impairment in the ageing vasculature. Nature Reviews Cardiology, 15, 555–565. 10.1038/s41569-018-0030-z PMC6612360 29795441

[phy271120-bib-0059] Vassalle, C. , Sciarrino, R. , Bianchi, S. , Battaglia, D. , Mercuri, A. , & Maffei, S. (2012). Sex‐related differences in association of oxidative stress status with coronary artery disease. Fertility and Sterility, 97, 414–419.e2. 10.1016/j.fertnstert.2011.11.045 22196713

[phy271120-bib-0060] Verbree, J. , Bronzwaer, A.‐S. G. T. , Ghariq, E. , Versluis, M. J. , Daemen, M. J. A. P. , van Buchem, M. A. , Dahan, A. , van Lieshout, J. J. , & van Osch, M. J. P. (2014). Assessment of middle cerebral artery diameter during hypocapnia and hypercapnia in humans using ultra‐high‐field MRI. Journal of Applied Physiology, 117, 1084–1089. 10.1152/japplphysiol.00651.2014 25190741

[phy271120-bib-0061] Verhaegen, D. , Smits, K. , Osório, N. , & Caseiro, A. (2022). Oxidative stress in relation to aging and exercise. Encyclopedia, 2, 1545–1558. 10.3390/encyclopedia2030105

[phy271120-bib-0062] Walker, A. E. , Kaplon, R. E. , Lucking, S. M. S. , Russell‐Nowlan, M. J. , Eckel, R. H. , & Seals, D. R. (2012). Fenofibrate improves vascular endothelial function by reducing oxidative stress while increasing endothelial nitric oxide synthase in healthy normolipidemic older adults. Hypertension, 60, 1517–1523. 10.1161/HYPERTENSIONAHA.112.203661 PMC3684180 23108655

[phy271120-bib-0063] Wang, C. , Reid, G. , Mackay, C. E. , Hayes, G. , Bulte, D. P. , & Suri, S. (2023). A systematic review of the association between dementia risk factors and cerebrovascular reactivity. Neuroscience & Biobehavioral Reviews, 148, 105140. 10.1016/j.neubiorev.2023.105140 36944391

[phy271120-bib-0064] Weijs, R. W. J. , Shkredova, D. A. , Brekelmans, A. C. M. , Thijssen, D. H. J. , & Claassen, J. A. H. R. (2023). Longitudinal changes in cerebral blood flow and their relation with cognitive decline in patients with dementia: Current knowledge and future directions. Alzheimer's & Dementia, 19, 532–548. 10.1002/alz.12666 35485906

[phy271120-bib-0065] Weintraub, S. , Dikmen, S. S. , Heaton, R. K. , Tulsky, D. S. , Zelazo, P. D. , Bauer, P. J. , Carlozzi, N. E. , Slotkin, J. , Blitz, D. , Wallner‐Allen, K. , Fox, N. A. , Beaumont, J. L. , Mungas, D. , Nowinski, C. J. , Richler, J. , Deocampo, J. A. , Anderson, J. E. , Manly, J. J. , Borosh, B. , … Gershon, R. C. (2013). Cognition assessment using the NIH toolbox. Neurology, 80, S54–S64. 10.1212/WNL.0b013e3182872ded PMC3662346 23479546

[phy271120-bib-0066] Wolters, F. J. , Zonneveld, H. I. , Hofman, A. , van der Lugt, A. , Koudstaal, P. J. , Vernooij, M. W. , & Ikram, M. A. (2017). Cerebral perfusion and the risk of dementia. Circulation, 136, 719–728. 10.1161/CIRCULATIONAHA.117.027448 28588075

[phy271120-bib-0067] Xing, C.‐Y. , Tarumi, T. , Liu, J. , Zhang, Y. , Turner, M. , Riley, J. , Tinajero, C. D. , Yuan, L.‐J. , & Zhang, R. (2017). Distribution of cardiac output to the brain across the adult lifespan. Journal of Cerebral Blood Flow and Metabolism, 37, 2848–2856. 10.1177/0271678X16676826 PMC5536794 27789785

[phy271120-bib-0068] Yazici, B. , Erdoğmuş, B. , & Tugay, A. (2005). Cerebral blood flow measurements of the extracranial carotid and vertebral arteries with doppler ultrasonography in healthy adults. Diagnostic and Interventional Radiology, 11, 195–198. 16320223

[phy271120-bib-0069] Zarrinkoob, L. , Ambarki, K. , Wåhlin, A. , Birgander, R. , Eklund, A. , & Malm, J. (2015). Blood flow distribution in cerebral arteries. Journal of Cerebral Blood Flow and Metabolism, 35, 648–654. 10.1038/jcbfm.2014.241 PMC4420884 25564234

[phy271120-bib-0070] Zhang, N. , Gordon, M. L. , Ma, Y. , Chi, B. , Gomar, J. J. , Peng, S. , Kingsley, P. B. , Eidelberg, D. , & Goldberg, T. E. (2018). The age‐related perfusion pattern measured with arterial spin labeling MRI in healthy subjects. Frontiers in Aging Neuroscience, 10, 214. 10.3389/fnagi.2018.00214 PMC6056623 30065646

[phy271120-bib-0071] Zuj, K. A. , Arbeille, P. P. P. , Shoemaker, J. K. , Blaber, A. P. , Greaves, D. K. , Xu, D. , Hughson, R. L. , & Ontario, W. (2012). Impaired cerebrovascular autoregulation and reduced CO2 reactivity after long duration spaceflight. AJP: Heart and Circulatory Physiology, 302, H2592–H2598. 10.1152/ajpheart.00029.2012 22492717

