# Supplementary material for: Supraphysiological ascorbic acid infusion attenuates cerebrovascular dysfunction in middle‐aged and older adults
Source: Physiol Rep. 2026 Oct 4;14(19):e71120. doi: 10.14814/phy2.71120 (PMC13635807; doi:10.14814/phy2.71120)
Supplement: Supplementary file 1 [file PHY2-14-e71120-s001.docx]

**Supraphysiological ascorbic acid infusion attenuates cerebrovascular dysfunction in middle-aged and older adults**

Theodore M. DeConne, Faria Sanjana, Elizabeth M. Habash, Kevin P. Decker, Joshua C Hobson, Wendy C. Nichols, Nicholas A. Rizzi, and Christopher R Martens.

**Supplemental Materials**

| **Supplemental Table 1. File-level analytic counts for transient hypercapnia stimulus.** | | | | |
| --- | --- | --- | --- | --- |
|  | **SAL** | | **AA** | |
|  | **Pre** | **Post** | **Pre** | **Post** |
| *Original files,* $N$ | 25 | 25 | 25 | 25 |
| Poor image quality, $N$ | 12 | 10 | 10 | 11 |
| P_ET_CO_2_ increase of ≥6 mmHg not achieved, $N$ | 0 | 0 | 2 | 0 |
| No file available, $N$ | 0 | 2 | 0 | 2 |
| Paired file not available (pre or post), $N$ | 2 | 2 | 3 | 2 |
| *Files remaining for analysis,* $N$ | 11 | 11 | 10 | 10 |
| **Supplemental Table 1.** **File-level analytic counts for transient hypercapnia stimulus.** Participant files were removed if 1) the file was not analyzable by Cardiovascular Suite; 2) a P_ET_CO_2_ increase of ≥6 mmHg not achieved; 3) the file was not available (e.g., participant dropped out after the first experimental visit); or 4) the paired pre- or post-infusion file for that infusion was not available. **Abbreviations:** CVR, cerebrovascular reactivity; P_ET_CO_2_, partial pressure of end-tidal CO2; SAL, saline infusion; AA, ascorbic acid infusion; pre, pre-infusion; & post, post-infusion. | | | | |

| **Supplemental Table 2. File-level analytic counts for sustained hypercapnia stimulus.** | | | | |
| --- | --- | --- | --- | --- |
|  | **SAL** | | **AA** | |
|  | **Pre** | **Post** | **Pre** | **Post** |
| *Original files,* $N$ | 28 | 28 | 28 | 28 |
| P_ET_CO_2_ increase of ≥6 mmHg not achieved, $N$ | 3 | 1 | 1 | 2 |
| No file available, $N$ | 3 | 4 | 2 | 6 |
| Paired file not available (pre or post), $N$ | 2 | 3 | 8 | 3 |
| *Files remaining for analysis,* $N$ | 20 | 20 | 17 | 17 |
| **Supplemental Table 2. File level analytic counts for sustained hypercapnia stimulus.** Participant files were removed if 1) a P_ET_CO_2_ increase of ≥6 mmHg not achieved; 2) the file was not available (e.g., participant dropped out after the first experimental visit); or 3) if the paired pre- or post-infusion file for that infusion was not available. **Abbreviations:** CVR, cerebrovascular reactivity; P_ET_CO_2_, partial pressure of end-tidal CO2; SAL, saline infusion; AA, ascorbic acid infusion; pre, pre-infusion; & post, post-infusion. | | | | |

| **Supplemental Table 3. Participant-level final characteristics** | | | | |
| --- | --- | --- | --- | --- |
|  | **Y** | **MA/O** | **P-value** |  |
| N | 9 | 19 | - |  |
| Age, years | 25 ± 2 | 65 ± 6 | <0.0001* |  |
| Man/Woman (%) | 5/4 (56/44) | 8/11 (42/58) | - |  |
| Height, cm | 169.4 ± 11.7 | 166.6 ± 10.2 | 0.55 |  |
| Weight, kg | 66.4 ± 13.7 | 77.9 ± 15.1 | 0.097 |  |
| BMI, kg/m^2^ | 22.89 ± 2.42 | 28.0 ± 4.4 | 0.0014* |  |
| FFM all, kg | 50.6 ± 12.4 | 51.1 ± 11.6 | 0.85 |  |
| FFM DXA, kg | 50.6 ± 12.4 | 48.8 ± 10.8 | 0.88 |  |
| FFM BIA, kg | NA | 57.6 ± 12.4 | 0.16† |  |
| NIH Toolbox Total Composite Fully Corrected T-Score | 57 ± 7 | 56 ± 7 | 0.54 |  |
| CVLT III Total Recall, Index Score | 108 ± 11 | 105 ± 17 | 0.55 |  |
| Resting SBP, mmHg | 106 ± 12 | 119 ± 14 | 0.062 |  |
| Resting DBP, mmHg | 65 ± 8 | 72 ± 9 | 0.058 |  |
| Resting MAP, mmHg | 78 ± 9 | 88 ± 10 | 0.038* |  |
| Resting PP, mmHg | 41 ± 9 | 46 ± 7 | 0.17 |  |
| Resting HR, bpm | 56 ± 7 | 62 ± 8 | 0.066 |  |
| Fasting TC, mg/dL | 188.1 ± 35.9 | 195.3 ± 42.6 | 0.73 |  |
| Fasting HDL-C, mg/dL | 64.6 ± 12 | 60.0 ± 11.4 | 0.40 |  |
| Fasting LDL-C, mg/dL | 104.1 ± 27.1 | 114.7 ± 33.2 | 0.54 |  |
| Fasting TG, mg/dL | 97.1 ± 50.3 | 101.8 ± 39.9 | 0.67 |  |
| Fasting Non-HDL-C, mg/dL | 123.6 ± 31.4 | 134.8 ± 33.9 | 0.52 |  |
| Fasting Blood glucose, mg/dL | 85.7 ± 7.4 | 93.8 ± 8.4 | 0.058 |  |
| **Supplemental Table 3.**  **Participant-level final characteristics.** Data are presented as mean ± SD. Rank differences between groups were analyzed using unpaired Mann-Whitney tests. Significance was set at p<0.05 and denoted by *differences between Y vs. MA/O and †differences between DXA and BIA within the same age group. FFM was measured using only DXA in Y adults. **Abbreviations:** Y, young adults; MA/O, middle-aged and older adults; min, minimum; max, maximum; FFM, fat free mass; DXA, dual x-ray absorptiometry; BIA, bioelectrical impedance; SBP, systolic blood pressure; DBP, diastolic blood pressure; MAP, mean arterial pressure; PP, pulse pressure; HR, heart rate; TC, total cholesterol; HDL-C, high-density lipoprotein-cholesterol; LDL-C, low density lipoprotein-cholesterol; TG, triglycerides; and Non-HDL-C, non-HDL-cholesterol. | | | | |

| **Supplemental Table 4. AA did not alter TCBF in Y or MA/O adults** | | | | | | | | |
| --- | --- | --- | --- | --- | --- | --- | --- | --- |
| **Variable** | **SAL** | | | | **AA** | | | |
|  | **Y adults** | | **MA/O adults** | | **Y adults** | | **MA/O adults** | |
|  | **Pre** | **Post** | **Pre** | **Post** | **Pre** | **Post** | **Pre** | **Post** |
| **TCBF, mL/min** | 1014.56 ± 223.36 | 1109.48 ± 166.83 | 1031.18 ± 131.36 | 1550.51 ± 943.87 | 1095.67 ± 288.95 | 1219.03 ± 313.47 | **1740.81 ± 812.46†** | 1642.79 ± 780.51 |
| **ICA flow, mL/min** | 387.42 ± 104.48 | 437.75 ± 92.31 | 532.38 ± 265.89 | 620.16 ± 434.05 | 414.03 ± 116.93 | 488.04 ± 153.63 | 675.81 ± 391.88 | 674.73 ± 381.08 |
| **ICA diameter, mm** | 4.77 ± 0.61 | 4.91 ± 0.47 | **5.99 ± 1.31†** | 6.00 ± 1.46 | 5.01 ± 0.50 | 5.17 ± 1.01 | 5.82 ± 1.49 | 5.80 ± 1.36 |
| **VA flow, mL/min** | 119.87 ± 25.99 | 116.99 ± 32.06 | 156.59 ± 98.65 | 157.77 ± 102.73 | 133.80 ± 43.56 | 121.47 ± 17.58 | 174.46 ± 88.49 | 150.14 ± 77.14 |
| **VA diameter, mm** | 3.55 ± 0.21 | 3.55 ± 0.16 | 3.89 ± 1.13 | 3.87 ± 1.05 | 3.66 ± 0.23 | 3.65 ± 0.27 | 4.03 ± 0.90 | 3.71 ± 0.84 |
| **Supplemental Table 4. AA did not alter TCBF in Y or MA/O adults.** Basal ICA blood flow and diameter, VA blood flow and diameter, and TCBF before and after the infusion of AA or SAL were assessed in Y and MA/O adults using uncorrected Fisher’s Least Significant Difference tests. **Abbreviations:** ICA, internal carotid artery; VA, vertebral artery; TCBF, total cerebral blood flow; Y, young adults; MA/O, middle-aged and older adults; SAL, saline infusion; AA, ascorbic acid infusion; pre, pre-infusion; and post, post-infusion. Statistical significance was set at P<0.05 and displayed in bold text. *Pre vs. post-infusion and †pre-infusion Y vs. MA/O adults. | | | | | | | | |

| **Supplemental Table 5. AA did not alter FMD in Y or MA/O adults** | | | | | | | | |
| --- | --- | --- | --- | --- | --- | --- | --- | --- |
| **Variable** | **SAL** | | | | **AA** | | | |
|  | **Y adults** | | **MA/O adults** | | **Y adults** | | **MA/O adults** | |
|  | **Pre** | **Post** | **Pre** | **Post** | **Pre** | **Post** | **Pre** | **Post** |
| **FMD, %** | 8.40 ± 1.82 | 8.28 ± 1.31 | **5.84 ± 2.32†** | 7.12 ± 2.17 | 6.99 ± 1.85 | 7.66 ± 1.49 | **4.96 ± 1.90†** | 6.45 ± 1.97 |
| **FMD, mm** | 0.31 ± 0.05 | 0.31 ± 0.06 | **0.22 ± 0.11†** | 0.26 ± 0.08 | 0.26 ± 0.077 | 0.27 ± 0.065 | 0.19 ± 0.062 | 0.23 ± 0.068 |
| **FMD, %/sec^-1^** | 0.000298 ± 0.000119 | 0.000303 ± 0.000098 | 0.000306 ± 0.000243 | 0.000302 ± 0.000177 | 0.000443 ± 0.000112 | 0.000401 ± 0.000119 | **0.000231 ± 0.000127†** | 0.000308 ± 0.000187 |
| **FMD, mm/sec^-1^** | 0.0000120 ± 0.0000064 | 0.0000120 ± 0.0000055 | 0.0000125 ± 0.0000128 | 0.0000113 ± 0.0000091 | 0.0000161 ± 0.0000034 | 0.0000151 ± 0.0000065 | **0.0000092 ± 0.0000052†** | 0.0000112 ± 0.0000067 |
| **Blood Flow at Peak, mL/min** | 10419.95 ± 1353.01 | 10269.71 ± 3137.94 | 8238.38 ± 3746.04 | 13202.79 ± 14085.72 | 5762.50 ± 3441.36 | 5812.37 ± 2007.69 | 9519.74 ± 8216.43 | 8202.32 ± 6340.85 |
| **Shear Rate at Peak, sec^-1^** | 35235.75 ± 22690.78 | 32231.80 ± 18691.05 | 26351.33 ± 12422.7 | 29048.30 ± 12890.26 | 16194.44 ± 3215.389 | 21167.44 ± 9758.223 | 25896.51 ± 12077.63 | 25177.77 ± 9691.388 |
| **Time to Peak, sec** | 65.83 ± 26.53 | 57.61 ± 23.18 | 56.22 ± 20.09 | 55.23 ± 17.63 | 43.69 ± 11.51 | 49.44 ± 12.10 | 58.17 ± 19.35 | 53.20 ± 11.79 |
| **Supplemental Table 5. AA did not alter FMD in Y or MA/O adults.** Differences in FMD measured before and after the infusion of AA or SAL in Y and MA/O adults were assessed using uncorrected Fisher’s Least Significant Difference tests. **Abbreviations:** FMD, flow mediated dilation; PCT, percentage; ABS, absolute; Y, young adults; MA/O, middle-aged and older adults; SAL, saline infusion; AA, ascorbic acid infusion; pre, pre-infusion; and post, post-infusion. Statistical significance was set at P<0.05 and displayed in bold text. *Pre vs. post-infusion and **†**pre-infusion Y vs. MA/O adults. | | | | | | | | |

**Supplemental Figure 1.** **Protocol figure.** Y (18-29 years) and MA/O (55-79 years) community-dwelling adults were enrolled in a placebo-controlled, randomized, crossover, intervention study. SAL (blue) and AA (yellow) infusions were used to “pharmaco-dissect” the effect of aging on cerebrovascular and peripheral vascular function. A minimum 4-day washout separated each infusion visit. FMD, TCBF, ICA CVR in response to transient hypercapnia, and MCA CVR in response to sustained hypercapnia were assessed before and after each infusion. Participants received a bolus infusion followed by a drip infusion that was sustained until all post-infusion vascular measurements were made. Abbreviations: Y, young; MA/O, middle-aged and older; SAL, saline infusion; AA, ascorbic acid infusion; FMD, flow mediated dilation; TCBF, total cerebral blood flow; MCA, middle cerebral artery; CVR, cerebrovascular reactivity; and ICA, internal carotid artery.

**Supplemental Figure 2.** **Differences between transcranial Doppler depth and power.** The transcranial Doppler (A) depth and (B) power used to insonate the middle cerebral artery were assessed before and after each infusion using Mixed-effects models. Data are presented as mean ± SD. Abbreviations: TCD, transcranial Doppler; SAL, saline; and AA, ascorbic acid.

**Supplemental Figure 3.** **The effect of AA infusion on serum concentrations.** Serum AA was assessed in (A) Y and MA/O adults before and after the infusion of AA and in (B) MA/O adults before and after the infusion of SAL. Serum AA measured during the SAL visit is only presented descriptively, given the small sample size. Pre-infusion means are displayed in light color and post-infusion means are displayed in dark color. Individual data points from Y and MA/O adults are displayed as black triangles and circles, respectively. Data are presented as mean ± SD. Abbreviations: SAL, saline; AA, ascorbic acid; Y, young adults; and MA/O, middle-aged and older.
